# Draft Genome Sequence of Kosakonia radicincitans UYSO10, an Endophytic Plant Growth-Promoting Bacterium of Sugarcane (*Saccharum officinarum*)

**DOI:** 10.1128/MRA.01000-19

**Published:** 2019-10-24

**Authors:** Martín Beracochea, Cecilia Taulé, Federico Battistoni

**Affiliations:** a Departamento de Bioquímica y Genómica Microbianas, Instituto de Investigaciones Biológicas Clemente Estable, Montevideo, Uruguay; Queens College

## Abstract

Kosakonia radicincitas UYSO10 is an endophytic bacterium that was isolated from stem tissues of Saccharum officinarum plants cultivated in Uruguay. UYSO10 is a diazotrophic indoleacetic acid-producing bacterium with growth-promoting effects on sugarcane. Here, we report the draft genome sequence, in which genes that are probably involved in the plant-bacterium interaction were identified.

## ANNOUNCEMENT

Mutualistic and symbiotic associations between plants and growth-promoting bacteria play an important role in plant health (1). In recent years, interest in strains belonging to the Kosakonia genus and their interactions with plants has increased. Thus, there are several reports of plant growth promotion by *Kosakonia* strains under greenhouse and field conditions (2–4). Strain UYSO10 was isolated from surface-sterilized stem tissues of Sacharum officinarum cv. FAM8177 cultivated in Uruguay, on LGI-P semisolid medium at 30°C, as described by Taulé et al. (5). Strain UYSO10 showed plant growth promotion effects upon inoculation onto micropropagated and setts of sugarcane cultivar LCP85384 under greenhouse conditions (6, 7). *In vitro* analysis showed that it is capable of producing indoleacetic acid to fix N_2_ and that it has endoglucanase activity (5, 6). Moreover, the strain was defined as a true endophyte of sugarcane plants (6). Genomics and functional analysis of the genes involved in biological nitrogen fixation (BNF) showed that the UYSO10 genome harbors two functional nitrogenases and that the inactivation of both nitrogenase-encoding genes diminishes its capacity for promoting plant growth (7).

For DNA extraction, a single colony of strain UYSO10 was taken from a frozen stock and grown in 10 ml of tryptic soy broth (TSB) medium at 28°C under agitation. Genomic DNA was extracted using the DNA purification kit Quick-gDNA fungal/bacterial miniprep kit (Zymo Research, USA). Single-end DNA libraries were prepared for sequencing with an Ion Torrent Personal Genome Machine (PGM) platform. A total of 658,146 single-end reads were generated, with an average read length of 300 bp and a coverage of 42×. The read quality was assessed with FastQC version 0.11.6 (8) and filtered with Sickle version 1.33 (9). *De novo* assembly was performed using SPAdes genome assembler version 3.11.1 with the parameters [-only-assembler] [-iontorrent] y [k = 21, 33, 55, 71] (10). The obtained genome sequence included 99 contigs (≥1,000 bp) with an *N*_50_ value of 346,125 bp and a GC content of 54%. The genome was uploaded to the Rapid Annotations using Subsystems Technology (RAST) version 2.0 annotation server under the classic annotation scheme (11). The UYSO10 annotation showed that it has 5,948 coding sequences and 1 copy of the rRNA operon. UYSO10 is most closely related to Kosakonia radicincitans DSM 16656^T^ (12), with which it shares 99% average nucleotide identity (ANI); this was determined using the Web service JSpeciesWS (13). Default parameters were used for all software unless otherwise specified. The K. radicincitans UYSO10 genome presents several genes related to plant colonization and plant growth promotion, including genes for flagellar and twitching motility, siderophore production, auxin biosynthesis, and production of volatile acetoin and 2,3-butanediol, as well as genes for nitrogen fixation (complete *nif* regulon and *anf* operon). Furthermore, it contains genes involved in the biosynthesis of type I, IV, V, and VI secretion systems.

### Data availability.

The raw sequence data of K. radicincitans UYSO10 have been deposited in the European Nucleotide Archive (ENA) under BioProject number PRJEB32347 and accession numbers ERR3365598 and ERR3367514. The assembled and annotated set of contigs is publicly available under assembly accession number GCA_902498885.
